# Visual Prosthesis: Interfacing Stimulating Electrodes with Retinal Neurons to Restore Vision

**DOI:** 10.3389/fnins.2017.00620

**Published:** 2017-11-14

**Authors:** Alejandro Barriga-Rivera, Lilach Bareket, Josef Goding, Ulises A. Aregueta-Robles, Gregg J. Suaning

**Affiliations:** ^1^Graduate School of Biomedical Engineering, University of New South Wales, Sydney, NSW, Australia; ^2^Faculty of Engineering and Information Technologies, University of Sydney, Sydney, NSW, Australia; ^3^Division of Neuroscience, University Pablo de Olavide, Sevilla, Spain; ^4^Department of Bioengineering, Imperial College London, London, United Kingdom

**Keywords:** visual prosthesis, retinal neurostimulation, conducting polymers, carbon nanotubes, nanocrystalline diamonds, silicon nanowires, quantum dots, living electrodes

## Abstract

The bypassing of degenerated photoreceptors using retinal neurostimulators is helping the blind to recover functional vision. Researchers are investigating new ways to improve visual percepts elicited by these means as the vision produced by these early devices remain rudimentary. However, several factors are hampering the progression of bionic technologies: the charge injection limits of metallic electrodes, the mechanical mismatch between excitable tissue and the stimulating elements, neural and electric crosstalk, the physical size of the implanted devices, and the inability to selectively activate different types of retinal neurons. Electrochemical and mechanical limitations are being addressed by the application of electromaterials such as conducting polymers, carbon nanotubes and nanocrystalline diamonds, among other biomaterials, to electrical neuromodulation. In addition, the use of synthetic hydrogels and cell-laden biomaterials is promising better interfaces, as it opens a door to establishing synaptic connections between the electrode material and the excitable cells. Finally, new electrostimulation approaches relying on the use of high-frequency stimulation and field overlapping techniques are being developed to better replicate the neural code of the retina. All these elements combined will bring bionic vision beyond its present state and into the realm of a viable, mainstream therapy for vision loss.

## Introduction

The number of blind people in the world is currently around 30 million (Stevens et al., 2013). Blindness is one of the most demoralizing and debilitating disorders as quality of life is seriously deteriorated. Apart from the obvious physical limitations such as reduced mobility, from a psychological perspective, there are numerous consequences directly related to the lack of the sense of sight. These include insomnia, social isolation, or even suicidal thoughts (Moschos, 2014). While some of the degenerative conditions of the eye can be prevented or treated, others such as retinitis pigmentosa have no cure. This group of diseases in which the neural network of the retina is affected is being targeted by researchers worldwide using a number of approaches.

Stem cell transplants are a promising therapy for the restoration of degenerated photoreceptors (Ramsden et al., 2013; Chader and Young, 2016). As stem cells have the ability to differentiate into other cell types, they can potentially be used to reconstruct the damaged retina. However, there remain a number of challenges to be overcome before stem cells may become a mainstream therapy for the restoration of vision. These include means of promoting a controlled laminar growth within the retina, and overcoming ethical concerns about the use of some types of stem cells.

Although, still far from reaching the patient, *in vivo* gene editing through the Clustered Regularly Interspaced Short Palindromic Repeat (known as CRISPR/Cas9) technique is also promising a treatment for those conditions with a genetic disorder resulting in vision loss (Bakondi et al., 2016; Suzuki et al., 2016). This technique allows now for knocking in DNA material in both, dividing and non-dividing cells *in vivo*, thus opening new possibilities to treat genetic retinal degeneration. This approach has been demonstrated effective in Royal College of Surgeons (RCS) rats and in S334ter-3 rats, both models of retinal degeneration. Yet, this therapy may not be realized clinically for a number of years or perhaps decades, as there are arduous testing requirements to demonstrate safety.

A third strategy is the optogenetic restoration of sight, which relies on the use of adeno-associated viral vectors carrying genes which encode light-sensitive proteins (Gaub et al., 2015). These vectors can impart light sensitivity to the remaining retinal neurons thus restoring some degree of visual perception. However, this approach currently lacks sufficient light sensitivity and therefore may provide limited visual benefit with a relevant potential for inducing immune responses (Busskamp et al., 2012).

**Figure 1:**
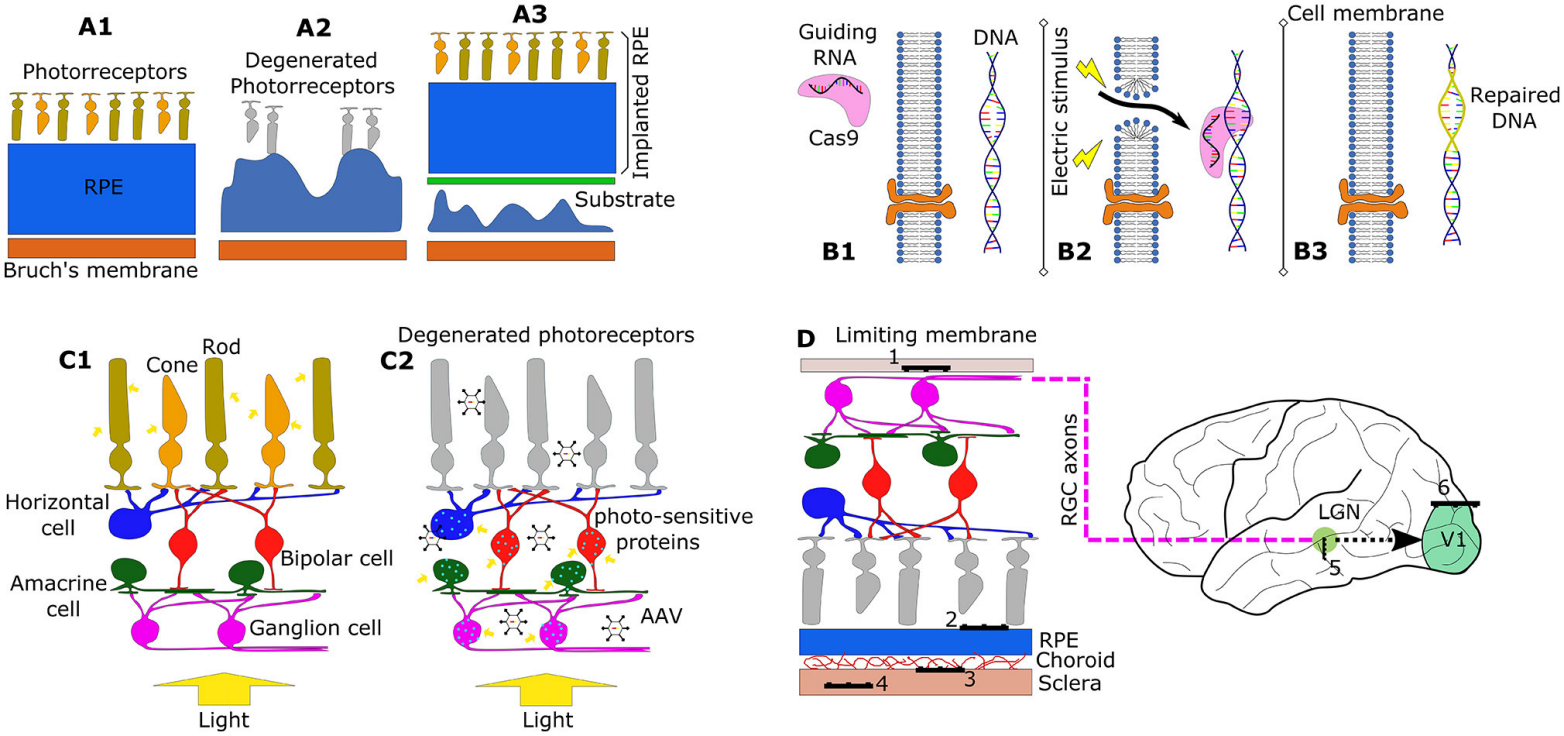
Schematic representation of different techniques to restore vision. **(A1–A3)** Illustrate the stem cell transplantation technique. **(A1)** Shows a healthy structure of the eye with healthy retinal pigmented epithelium (RPE). **(A2)** Represents the degeneration of the retina due to a degradation of the RPE, including loss of photoreceptors. **(A3)** illustrates an example of an implant of a RPE patch graft of stem cells using a supporting substrate. **(B1–B3)** describes the CRISPR/Cas9 genome editing technique. **(B1)** Shows the delivery of RNA-guided Cas9 nuclease to the extracellular space of the retinal tissue. **(B2)** Illustrates how electroporation (change in cell membrane permeability using an electric field) can allow Cas9 to enter to the cytoplasm of the cells. Cas9 then interrogates the cell's DNA allowing repair of the target gene **(B3)**. **(C1,C2)** Illustrate the mechanism of sight restoration using optogenetics. In physiological function, light transduction takes place at the photoreceptors **(C1)**. When degradation occurs **(C2)**, an injection of adeno-asociated viral (AAV) vectors can induce expression of photosensitive proteins in the remaining retinal neurons. **(D)** Illustrates the implantation sites for electrode arrays in the restoration of visual function using electrostimulation: (1) epiretinal, (2) subretinal, (3) suprachoroidal, (4) intrascleral, (5) thalamic, and (6) cortical prostheses.

While these three biological approaches are providing hope for the blind, stimulation of the visual system is the only effective option that has reached clinical practice and that is currently providing a solution to restore at least some functional vision to a number of patients in various parts of the world (Barnes et al., 2016; da Cruz et al., 2016). An illustrative description of the aforementioned techniques is shown in Figure 1.

The normal retina presents a complex neural structure that encodes visual inputs through a series of graded membrane potentials that ultimately result in electrical signals known as action potentials (APs) that are elicited from retinal ganglion cells (RGCs). These cells project their axons through the optic nerve toward different visual center thus providing a link between the eye and the brain (Masland, 2012). In the degenerate retina, phototransduction is no longer possible as a consequence, visual perception is impeded. However, neurons within the retina can retain part of their function and are capable of responding to electrical stimuli. Bionic vision provides a connection between an image sensor, typically a camera, and the visual system by substituting lost photoreceptor function with electrical impulses that produce APs. Images are typically processed by an externally-worn computer which instructs the implanted system to deliver electrical impulses via implanted electrodes (Brandli et al., 2016). Various stimulation sites have been considered: the visual cortex (Lewis and Rosenfeld, 2016), the optic nerve (Lane et al., 2016), the lateral geniculate nucleus (Nguyen et al., 2016) and the retina (da Cruz et al., 2016).

Electrical stimulation of the retinal neurons offers two clear advantages over other approaches. From a safety perspective, the surgical risks associated with the implantation of the electrodes are considerably lower compared to other visual neurostimulators (Weiland et al., 2005). Moreover, neural responses elicited in the retina will propagate to the midbrain and on to the visual cortex in a way more similar to physiologically natural visual systems than other sites of intervention (Eiber et al., 2013). Retinal electrostimulation requires an array of electrodes to be implanted in the eye, in particular in the epiretinal (Duncan et al., 2017), subretinal (Stingl et al., 2015) or in the supra-choroidal space (Bareket et al., 2017), in order to activate the RGCs both directly and indirectly via the remaining network of retinal neurons.

## Problems and limitations

Traditionally, retinal stimulation has consisted of the delivery of constant-current pulses via implanted electrodes. Limitations in the charge-carrying capacity of commonly used electrode materials can lead to unwanted or unsafe electrochemical reactions during stimulus delivery (Morton et al., 1994). These reactions create compounds which may interact with biomolecules leading to neuronal death and limiting both the benefit and longevity of the implanted device. To avoid permanent chemical reactions from taking place, these devices typically rely on biphasic pulses carrying equal amounts of charge in each phase with opposite signs and of sufficiently short duration to avoid irreversible effects (Humayun et al., 1999). In the case of metallic electrodes, the exposed surface area is directly related to their electrochemical properties. In other words, the larger the area of the electrode, the less likely it is to degrade under conditions of electrical stimulation (Green et al., 2014). While visual prostheses designers seek to improve visual resolution by increasing the number of electrodes contained in a prosthetic array (Stingl et al., 2015), this requires their corresponding miniaturization which, in turn, increases the concerns relating to electrochemical reactions.

At the same time, there is a relationship between the minimum amount of charge required to elicit neural activation and the distance between the target neurons and the electrode (Jensen et al., 2003). As technology enables improved miniaturization of the electrodes, the electrochemical properties limit the amount of current and therefore determine the size beyond which activation is no longer possible without creating potentially damaging electrochemical reactions. Accordingly, electrode design and stimulation parameters are limited by the charge injection properties of the electrode material, which represent a constraint in the amount of charge that can be injected per unit of area. In an in vivo study of visual perception thresholds in a feline model, it was found that smooth platinum electrodes had charge injection limit (CIL) smaller than the threshold for vision perception (45 μC·cm^−2^ CIL for smooth platinum vs. 90 μC·cm^−2^ for effective stimulation) (Green et al., 2014). Laser roughening of the platinum electrodes did successfully increase the CIL to just above the perception thresholds at phase lengths of 0.2 ms and longer. A review of stimulation thresholds in humans revealed that only two of ten reported thresholds values are within the electrochemical limits of smooth platinum electrodes (Green et al., 2014). Although CIL can be improved by patterning the surface of the electrodes, the reduction in the electrode diameters achieved in the recent years contributes in the opposite direction leaving the problem unsolved.

Another challenge within the current technology is the so called “crosstalk” or interference between stimulating electrode sites. For instance, if the electrodes are densely packed, activation of an electrode at one site may have deleterious effects on neighboring sites (Abramian et al., 2014). Note that high-count electrode arrays also allow for the use of stimulation strategies that can mitigate the effects of electric crosstalk. Further, the geometrical convergence of passing axons of the RGCs toward the optic disc (FitzGibbon, 2017) presents an additional challenge to be overcome in avoiding activation of neurons whose cell bodies lie distal to the stimulating electrode's physical location. The combined consequence of these two factors leads to relatively complex visual percepts or phosphenes. While a punctate phosphene corresponding precisely with the location of the stimulating electrode is sought after, the result in practice can substantially differ to this ideal. While electrical return path configurations such as hexapolar or the quasi-monopolar stimulation paradigms provide more contained spread of the electric field using hexagonal guards to recover electric current (Matteucci et al., 2013), the combined effect of cross-talk and axonal stimulation has led to the reporting of very complex phosphene shapes in clinical trials (Nanduri et al., 2008; Horsager et al., 2011; Sinclair et al., 2016). Despite its contribution to complex phosphenes, electric crosstalk can be beneficial, as it may reduce activation thresholds due to field summation (Matteucci et al., 2016), or may inhibit neural activation as the effective overlap of electric fields bring the extracellular potential above the inhibition threshold (Barriga-Rivera et al., 2017).

The capacity to selectively stimulate different functional information pathways would potentially yield improved visual perceptions by restoring a more natural “neural code.” Exacerbating this objective is a reorganization of the neural circuitry within the retinal neural network during the course of retinal degenerative diseases (Jones et al., 2016). With electrical stimuli activating RGCs both directly and indirectly, and with a number of information pathways to the brain, replication of the neural code remains a substantial challenge. Although it has not been confirmed, some of the complexities reported in the visual percepts elicited by retinal neurostimulation could be explained in terms of the confounding messages being sent to the brain (Nanduri et al., 2008; Luo et al., 2016). An example of this contradictory information is the simultaneous activation of ON and OFF pathways produced by the stimulation pulses. This is akin to a light being both turned on and off at the same time. The visual sensation produced by this information being sent to the brain has been recreated computationally in an article by Fine and Boynton (2015).

## Strategies to improve bionic vision

New technologies are being developed to overcome some of the difficulties researchers and engineers are facing in the development of better visual prostheses. A combined approach that includes new biomaterials and novel neurostimulation approaches may ultimately lead to devices with greater integration with the remaining functional pathways of the visual system that can allow new stimulation paradigms to better replicate the natural neural code of the retina.

### Conducting polymers

**Figure 2:**
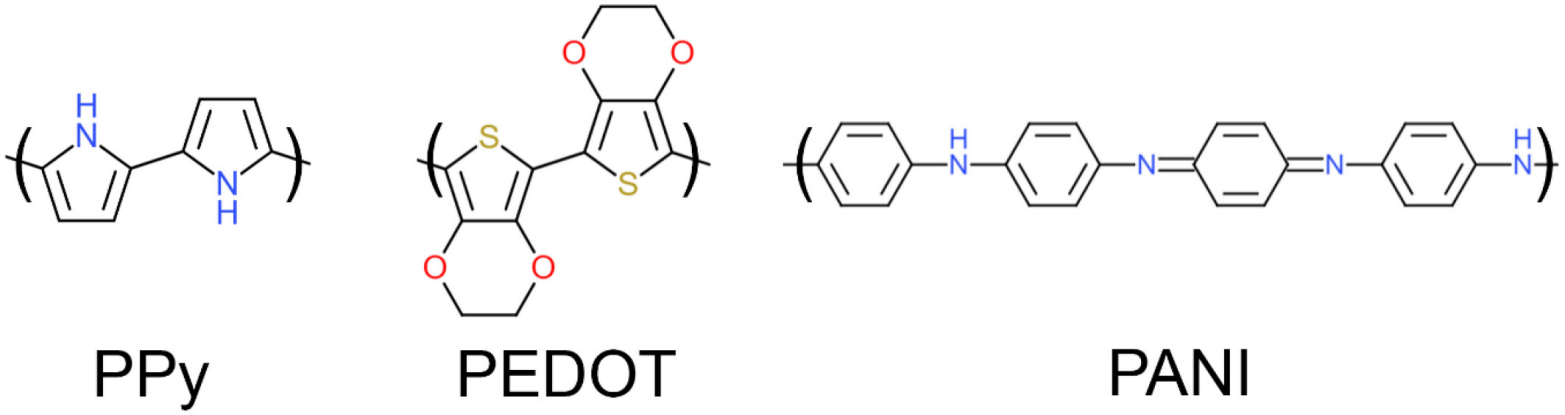
Chemical structures of polypyrrole (PPy), poly(3,4ethylenedioxythiophene) (PEDOT) and polyaniline (PANI).

Conducting polymers (CP) are a promising alternative to traditional electrode materials due to their low impedance and high CIL. CPs are a class of materials characterized by their conjugated backbone structure (alternating single and double bonds between adjacent carbon atoms) as shown in Figure 2.

**Figure 3:**
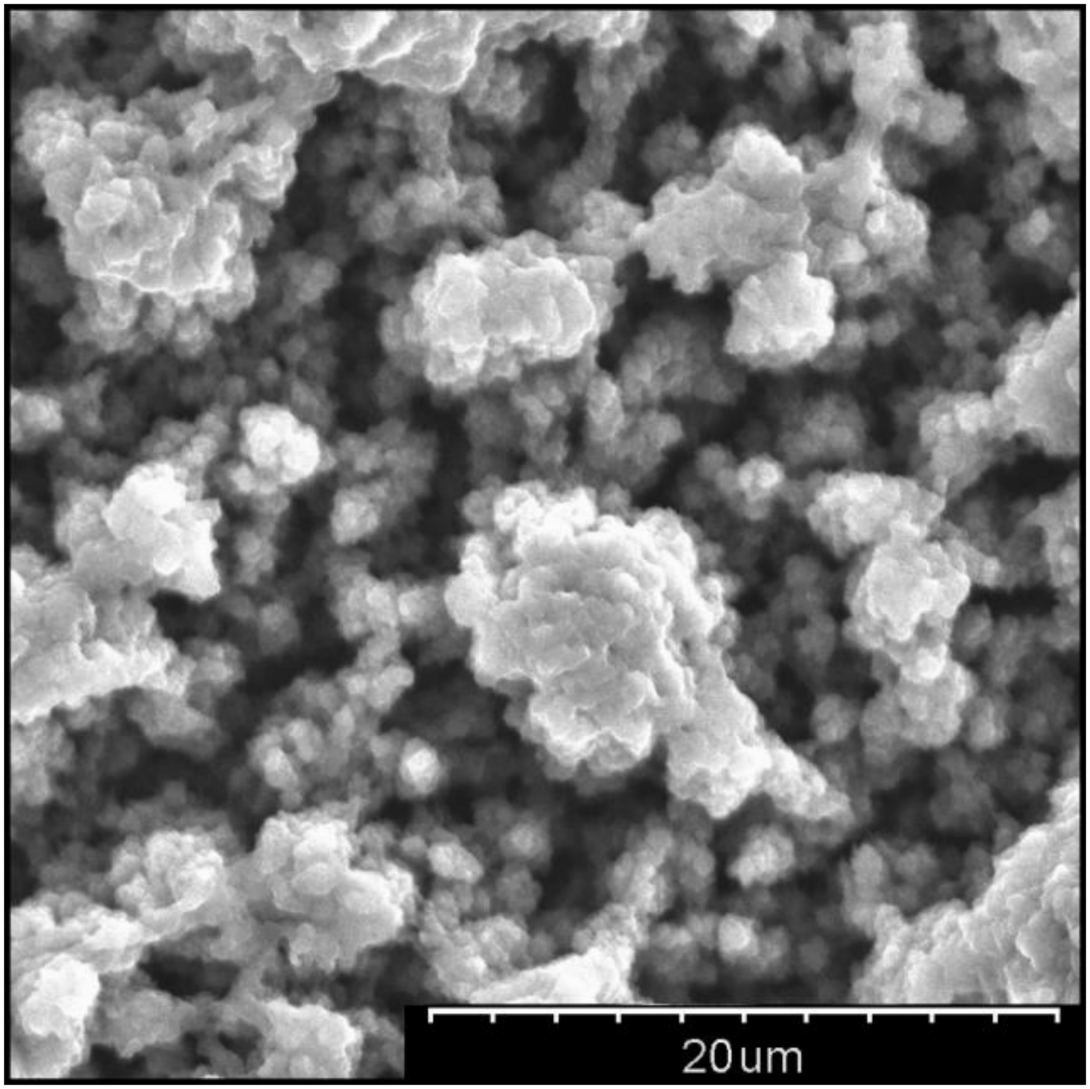
Scanning electron micrograph showing nodular surface morphology of PEDOT/pTS at 2500X magnification.

Each double bond along the conjugated backbone is comprised of a strong *sigma* bond and a delocalised *pi* bond. Repeated conjugation along the backbone gives rise to a conduction valence band which, when doped with an anionic dopant, facilitates charge conduction. The three main CPs studied for biomedical applications, shown in Figure 2, are polypyrrole (PPy), poly(3, 4-ethylenedioxythiophene) (PEDOT), polyaniline (PANI). These families of CPs are preferred over other formulations due to their demonstrated biological safety, chemical stability and electrochemical properties. The choice of dopant will also affect the properties of the resulting CP coating; poly(styrene sulfonate) (PSS), paratoluenesulfonate (pTS) and perchlorate ions (${\text{ClO}}_{4}^{-}$) are commonly used dopants for biomedical applications. CPs are typically electrochemically deposited onto an electrode substrate from a solution containing the CP monomer and an anionic dopant. Electrochemically deposited CPs have a highly nodular microstructure which acts to increase an electrode's topographical surface area. Figure 3 shows the surface morphology of PEDOT/pTS coated platinum electrode. The high surface index of CP coatings is partially responsible for their reduced impedance, increased charge storage capacity (CSC) and increased CIL. Coating platinum with PEDOT has been shown to decrease impedance by up to two orders of magnitude and increase CIL 15-fold at short phase lengths (from 0.08 mC·cm^−2^ for platinum to 1.2 mC·cm^−2^ for PEDOT at 100 μs) and 35-fold at longer phase lengths (from 0.12 mC·cm^−2^ for platinum to 3.9 mC·cm^−2^ for PEDOT at 800 μs) (Green et al., 2013a). Despite the promising performance of CP in *in vitro* testing, there has been limited success in translating this benefit to the *in vivo* setting. Implant studies have shown that coating an electrode with CP nanotubes can decrease electrode impedance by three orders of magnitude *in vitro*, however this benefit is reduced to only a 2-fold decrease in the *in vivo* setting (Abidian et al., 2009). The major factor determining *in vivo* performance is the biological response at the neural interface. Several approaches to overcoming this limitation have been investigated, including modifying the CP structure, creation of CP-based composites, and biofunctionalisation of CP materials.

#### Modified conducting polymer materials

Structuring of CPs on the nanoscale can be used to fundamentally alter the performance of the CP electrode coatings. Nanostructuring can take the form of nanoporosity, nanowires or nanotubes (Liu et al., 2008; Ghasemi-Mobarakeh et al., 2009; Xie et al., 2009; Kang et al., 2011). Nanoporosity has been used to increase the surface area of electrodes as well as modulate the drug-release and biosensing properties of CP systems (Luo and Cui, 2009a,b; Guo et al., 2011; Kang et al., 2011; Szultka-Mlynska et al., 2016). Nanoporosity is typically achieved by depositing around a template, such as polystyrene beads, which is removed after deposition. CP nanotubes have increased the CSC and decreased impedance compared to conventional CP coatings. CP nanotubes are typically fabricated by electrospinning or by electrodepositing CP around a nanotube template (Abidian et al., 2006, 2009; Liu et al., 2008; Ghasemi-Mobarakeh et al., 2009; Xie et al., 2009; Prabhakaran et al., 2011). Abidian et al. fabricated PEDOT and PPy nanotubes by electrochemically depositing CP around electrospun fibers of poly(lactic acid) and poly(lactic—co-glycolic acid) (Abidian et al., 2006, 2009, 2010). PEDOT nanotubes were found to have lower impedance and CSC (2.5 ± 1.4 kΩ at 1 kHz and 392 ± 6.2 mC·cm^−2^) compared to iridium (468.8 ± 13.3 kΩ at 1 kHz and 0.1 ± 0.5 mC·cm^−2^). When PEDOT nanotube electrodes were implanted in the barrel cortex of rats, they provided a stable reduction in impedance at 1 kHz over 49 days of implantation from 980 ± 15 kΩ for uncoated gold electrodes down to 521 ± 18 kΩ for PEDOT nanotube coated electrodes (Abidian et al., 2009).

While conducting polymers offer improved electrochemical properties compared to metallic electrodes, their mechanical properties present key limitations in the context of a chronic neuroprosthetic implant device. While conducting polymers are considerably softer than metals with moduli ranging 50 MPa to 8 GPa (Wang and Feng, 2002; Wang et al., 2009; Hassarati et al., 2014), they are still several orders of magnitude stiffer than neural tissue (0.5–1 kPa) and the retina (200–400 kPa). This mechanical mismatch is hypothesized to be a contributing factor to the biological inflammatory response to implantation which results in fibrotic encapsulation, increasing the impedance of the tissue-electrode interface and increasing the distance between the electrode surface and the target cells for electrical stimulation. Furthermore, conventional conducting polymers are brittle and prone to break-up and delamination. Studies have examined the long-term stability of CP coatings, examining accelerated aging, sterilization and long-term stimulation and found that while some CP coatings can be very stable under prolonged use (over 1.2 billion stimulation cycles) others are very dependent on the roughness of the underlying substrate and can fail in as little as 10 days (Boretius et al., 2011; Green et al., 2011, 2012a). This limitation is a considerable challenge to the implementation of CP-based technologies within chronically implanted devices and as such improving the mechanical robustness of CP-based coatings has become a focal point for ongoing research. One approach to overcome these mechanical limitations is the fabrication of CP-based composites, combining the electrochemical functionality CPs with the mechanical properties of softer materials such as hydrogels. Hydrogels are commonly used in tissue engineering because of their mechanical and structural similarity to soft tissue. Conducting hydrogels (CH) can be fabricated by electrochemically depositing a CP throughout a preformed hydrogel matrix, thus creating an interpenetrating network of the two polymer systems. Green et al. has demonstrated that this method can be used to create a composite electrode material that maintains the electrochemical benefits of conventional CP coatings while reducing the modulus from 40 MPa down to 2 MPa (Green et al., 2012b). The encapsulation of CP components within a soft hydrogel matrix also removes the problems of brittle failure and delamination associated with conventional CP coatings. Many CH systems have been reported, a summary is given below in Table 1.

**Table 1:** Summary of reported conducting hydrogel systems.

**Conducting polymer**	**Hydrogel**	**Summary**	**References**
PPy	Polyacrylamide	CH containing <5% PPy had similar CSC as conventional PPy	Kim et al., 2000
PANI	Poly((2-acrylamido-2-methyl propane sulphonic acid)	Electrical conductivity of 4 S.cm^−1^ achieved with high PANI loading	Siddhanta and Gangopadhyay, 2005
PANI	PANI-phytic acid	High capacitance electrodes (480 F.g^−1^) retaining 83% of original capacitance after 10,000 cycles	Pan et al., 2012
PANI	Poly(acrylic acid)	Mechanically strong gels (1.7 MPa fracture stress) with moderate conductivity (5 mS.cm^−1^)	Xia and Zhu, 2011
PEDOT	PVA-Taurine	High CSC (130 mc.cm^−2^), low impedance (24 Ω.cm^2^) electrodes	Goding et al., 2017b
PEDOT	PVA-Heparin	Reduce impedance of platinum electrodes by 85%. Biofunctionalisation to modulate biological response	Green et al., 2012b; Cheong et al., 2014

#### Application of conducting polymers to implantable devices

To date, there have been limited reports of the use of conducting polymer electrodes for *in vivo* stimulation applications. However, early reports have demonstrated that the use of CP-based electrodes greatly improves the performance of stimulating electrodes. A summary of *in vivo* testing of CP electrodes is given below in Table 2. While *in vitro* testing of CIL has shown CP-based systems to have a CIL of between 10 and 30 times greater than bare platinum (Green et al., 2012a, 2013a), this has yet to be reliably demonstrated in the *in vivo* setting.

**Table 2:** Summary of studies using conducting polymer electrodes in implantable devices for stimulation-based applications.

**Electrode**	**Implant model**	**Summary**	**References**
PEDOT/LiClO_4_ nanotubes	Barrel cortex of rat	PEDOT provided stable reduction in impedance of gold electrode by 46% during 49 days of implantation	Abidian et al., 2009
PEDOT/PSS-CNT	*Ex-vivo* retina from rat	Reduced stimulation thresholds compared to TiN electrode	Samba et al., 2015
PEDOT/pTS	Suprachoroidal implantation in feline	PEDOT had half the interfacial voltage of platinum control	Green et al., 2013a
PEDOT/PSS	Barrel cortex of rat	200 kΩ reduction in impedance and CIL 15 times higher compared to PtIr control	Venkatraman et al., 2011
PPy/pTS-NGF	Cochlea of guinea pig	Nerve growth factor delivered from PPy increased SGN density and reduced response threshold	Richardson et al., 2009

However, early results demonstrate PEDOT coatings reduces the transient and residual voltage across the electrode under biphasic stimulation, indicating the increase in CIL observed *in vitro* is carried over to the *in vivo* setting (Venkatraman et al., 2011; Green et al., 2013a). The increase in CIL conferred by CP coatings may ultimately allow for the reduction of electrode size without compromising device safety and efficacy. Furthermore, the reduced impedance of CP electrodes may allow devices to operate with reduced power requirements. The application of CP coatings to vision prosthesis devices may facilitate the development of arrays with smaller, more densely packed electrodes enabling improved device selectivity and resolution.

### Nanomaterials

Recent developments in materials and nano-engineering open new routes in interfacing with neurons that diverges from the traditional approach based on electrical stimulation through metal electrodes. Materials such as carbon nanotubes (CNTs) (Gabriel et al., 2009; Shoval et al., 2009; David-Pur et al., 2013, 2014; Eleftheriou et al., 2017), nanocrystalline diamonds (NCDs) (Hadjinicolaou et al., 2012; Ganesan et al., 2014; Ahnood et al., 2017), and silicon nanowires (Si NWs) (Khraiche et al., 2011, 2013; Ha et al., 2016) have attracted attention as promising candidates for improved electrical activation of neurons, and were demonstrated for activation of retinal neurons. These materials offer enhanced electrochemical properties and superior neuron-electrode mechanical attachment through unique surface topography and charge injection mechanism. Nanomaterials were also proposed for optical activation of neurons (Winter et al., 2001; Pappas et al., 2007; Bareket-Keren and Hanein, 2014) and applied to stimulate light-insensitive retinas (Bareket-Keren and Hanein, 2014).

#### Carbon nanotubes

CNTs offer several advantages over metal stimulating electrodes. Mainly, the combination of capacitive charge transfer mechanism and high electrochemical surface area, contributing to high charge injection capacity, high specific capacitance, and low interfacial impedance (Gabay et al., 2007; Bareket-Keren and Hanein, 2012; David-Pur et al., 2014). CNTs also provide an effective scaffold for neuronal growth and attachment, and it has been suggested that a mechanical mechanism of neurite entanglement facilitate this strong neuron-CNT affinity (Sorkin et al., 2008; Malarkey and Parpura, 2010; Voge and Stegemann, 2011). CNTs can be readily modified with different bioactive molecules (polymers, peptides, proteins) to improve their biocompatibility (Bekyarova et al., 2005; Bottini et al., 2011). Mattson et al. (2000) were the first to propose the use of CNTs as a substrate for neuronal growth. Since then extensive investigations revealed the suitability of CNTs to support neuronal growth and neurite branching (Mattson et al., 2000; Hu et al., 2004; Matsumoto et al., 2007; Cellot et al., 2009; Chao et al., 2009), to electrically interface with neurons (Gabay et al., 2005; Wang et al., 2006; Mazzatenta et al., 2007; Keefer et al., 2008; Shein et al., 2009; Bareket-Keren and Hanein, 2012), and further toward neural implants (Webster et al., 2003; Gabriel et al., 2009; Fuchsberger et al., 2011; David-Pur et al., 2014).

Recording of APs from RGCs of isolated rabbit retinas using CNT coated Pt electrodes was achieved by Gabriel, et al. (Gabriel et al., 2009). CNT coatings were easily achieved by drop casting CNT suspension onto an electrode and drying. Pt/CNT electrodes exhibited lower interfacial impedance and lower average noise in the recorded signals compared with uncoated Pt electrodes (Gabriel et al., 2009). While using drop casting is simple and useful for proof-of-concept *in vitro* studies, these coatings may not be suitable for long-term clinical applications due to poor adhesion of CNT to the metal substrate, in particular under sustained electrical stimulation.

Stronger adhesion can be formed by direct growth of CNTs directly onto TiN substrate using chemical vapor deposition (Gabay et al., 2007). Such pristine, high density, 3D CNT microelectrodes (60 and 30 μm in diameter) were used by Shoval et al. (2009) to record and stimulate RGC activity *in vitro*. Neonatal mouse retinas were mounted onto a CNT multi-electrode array (MEA), and a “Velcro” effect, where CNTs became tightly bound to the tissue was noticeable. Electrical recordings of spontaneous neuronal activity were consistently obtained, exhibiting typical bursting and propagating waves behavior. The SNR of the recorded signals was by up to three times higher compared to that of porous TiN electrodes. Moreover the amplitude of the signals gradually increased over the duration of the experiment (hours), implying an improvement in cell-to-electrode coupling (not observed with TiN arrays) (Shoval et al., 2009). Similar effects, as well as a gradual decrease in stimulation thresholds and an increase in cellular recruitment were observed in a later study by the same group (Eleftheriou et al., 2017). Here the investigators used explants of dystrophic mouse retinas (outer retina degeneration) mimicking the case of epiretinal implantation. The extracted retinas were flattened either on CNT electrodes or on isolated CNT islands and changes to the morphology of the bio-hybrid retina-CNT composite, and to the activity of ganglion cells were examined over a period of up to 3 days (Eleftheriou et al., 2017). Progressive integration of CNT structures into the inner retina without initiating gliosis response was observed. The authors noted that vitreous residues may compromise this effect. It was suggested that digestion of portions of the vitreous and of the internal limiting membrane prior to implantation of the electrodes using glycosidic enzymes could be applied in future bio-hybrid retinal arrays. This strong CNT-retina adhesion could potentially solve the fundamental problem with fixation of epiretinal arrays. Moreover, intracellular activation could be achieved by penetration of CNTs into individual RGCs, thus substantially lowering the amount of charge required for stimulation.

Flexible CNT MEAs, based entirely on CNT embedded in different polymeric supports (medical tape, PDMS, PI and Parylene) were also investigated to support mechanical compatibility with the tissue in contact. David-Pur et al. (2014) realized such flexible CNT arrays through a unique fabrication process based on growing loosely bound high-density CNT patterns on a silicon dioxide substrate, and transferring them directly onto a flexible support through a peel-off process (David-Pur et al., 2013, 2014). Embryonic chick retinas were flattened on these flexible arrays, with RGC layer facing down, as in an epi-retinal implant. Spontaneous activity waves were recorded and stimulation was achieved with a threshold of 4 nC. Validation of the synaptic processes was also demonstrated through silencing of RGC activation using synaptic blockers (David-Pur et al., 2014).

#### Nanocrystalline diamond

Nanodiamonds were also recently proposed as neuroelectrode material (Hadjinicolaou et al., 2012; Bendali et al., 2015; Ahnood et al., 2017). NCD becomes electrically conductive providing it is doped with nitrogen or trimethyl boron (Garrett et al., 2012; Kiran et al., 2012). Both boron and nitrogen doped diamond coatings show promising biocompatibility and suitability for neural stimulation neurons (Chen et al., 2010; Bendali et al., 2014). To increase the double layer capacitance and reduce the impedance, nitrogen doped ultra-nanocrystalline diamond (N-UNCD) electrodes were coated with Pt or EIROF, or alternatively activated via an electrochemical process (Garrett et al., 2012). For similar effects, boron doped diamond (BDD) electrodes were enhanced through a topographical modification by deposition of the NCD onto vertically aligned CNTs as a template interlayer, resulting with CIC of up to 10 mCcm^−2^ (measured *in vitro* in saline; Hébert et al., 2014; Piret et al., 2015).

Researchers from University of Melbourne have recently reported the development of an epiretinal system based on NCD technology. High-density monolithic integration of conductive N-UNCD directly onto insulating polycrystalline diamond enables construction of hermetic all-diamond arrays (Hadjinicolaou et al., 2012; Ganesan et al., 2014; Ahnood et al., 2017). The all-diamond electrode array consists of 256 microelectrodes (120 × 120 μm, and pitch of 150 μm) directly deposited onto a sheet of polycrystalline diamond (Ahnood et al., 2017). The authors noted that the fabrication process allows the pitch to be reduced by one order of magnitude (up to ~15 μm) potentially permitting thousands of electrodes within a 2 by 2 mm array. The Suitability of N-UNCD microelectrodes for stimulation of retinal neurons was assess *in vitro* using explanted rat retinas (Hadjinicolaou et al., 2012). APs were recorded from single ganglion cells adjacent to the stimulating channel using patch clamp (Hadjinicolaou et al., 2012). The biocompatibility of various forms of diamond was verified *in vitro* using cultures of rat cortical neurons (Tong et al., 2014) and *in vivo* when implanted into the back muscle of guinea pigs for 4–15 weeks (Garrett et al., 2016). Next, the compatibility of N-UNCD with retinal tissue was examined *in vivo*. Discs of 40 μm thick N-UNCD were subretinally implanted in the eyes of blind rat (model for RP) for 3 months. Fundus imaging and immunohistochemical studies showed that the retina tissue remained intact with no significant difference between sections examined away from the implant (control) and next to it, thus validating that N-UNCD is non-cytotoxic and biochemically stable for the duration of the study.

Increasing the resolution of retinal implants by using BDD/protein electrode coatings that specifically repel the adhesion and growth of glia cells while promoting the attachment of neurons was recently proposed by Bendali et al. (2014). Retinal implants are often in contact with a surface glial layer that may compromise the effective resolution of the device (Bendali et al., 2014). Neuronal electrodes are often end up being in contact with a surface sealing glial layer rather than with the neurons, leading to a gradual loss of function (Rousche and Normann, 1998; Maynard et al., 2000). This is the case for both retinal (specifically, epiretinal, and subretinal) and cortical visual implants. In the study by Bendali et al. (2014), adult retinal cell cultures were grown on either pristine of protein coated BDD electrodes. Glial cells growth was enhanced on the protein coated BDD whereas bipolar cells grew equally well on both pristine and coated BDD. In addition it was possible to engineer the adhesion of the protein to the BDD through different terminations either enhanced (Hydrogen-terminated) or prohibited (oxygen-terminated) protein coating. The authors suggested a design where a protein coated base will promote glial cell growth thus repelling them from the non-coated penetrating electrode tip (Bendali et al., 2014).

Next, flexible NCD electrode arrays were realized through a peel-off process using flexible substrates deposited on a 3D mold. The conducting material (either BDD or Pt) was deposited inside the groves formed by the molding process (Bendali et al., 2015). Three flexible prototypes were examined: polyimide, polyimide/BDD and polyimide/Pt. The implants were then inserted into the subretinal space of P23H rats, a model of retinal degeneration. Degenerated retinas could “mold” themselves into the inside of the wells, thereby isolating bipolar neurons for specific, independent stimulation (Bendali et al., 2015). The investigators further proposed a model for predicting the visual acuity that can be achieved using planar and 3D NCD implants with a distant ground or with a ground grid, thus demonstrating the potential of 3D designs for increasing the resolution of retinal implants (Bendali et al., 2015).

#### Optoelectronic silicon nanowires

A hybrid optoelectrical retinal prosthesis based on light sensing Si NWs is being developed by Silva et al. (Khraiche et al., 2011, 2013; Ha et al., 2016). The system comprises of an inductive telemetry link, stimulation pulse demodulator, charge-balancing series capacitor, and a subretinal Si NW electrode array. The system requires application of external power delivered by a single wireless inductive link. The NWs are designed to serve each as an electrode penetrating into retina. Two wires are used to connect the electrode array to the stimulator and to the nearby ground electrode. Upon application of voltage bias, the device produces a current pulse that scales with the intensity of light detected by the NWs. This hybrid optoelectronic system provides separate spatial (incidence light) and temporal (electrical bias) control over stimulation, as well as tuneable gain in case of lower light intensity thresholds. A distinct advantage of this architecture compared with other systems for electrical activation of the retina is that enhancing the resolution incurs almost no increase in hardware beyond the density of the electrode array. A proof of concept validation of the system was performed *ex vivo* by recording the activity from isolated retinal explants from P23H degenerated rats. A significant increase in neural response was observed under application of both bias and light. Pulsed light stimulation by itself (at low bias), or light off conditions with bias on, did not elicit a neural response, validating the feasibility of the proposed system.

#### Photoactive quantum dots based interfaces

An alternative approach to electrical stimulation is optical activation of retinal neurons through photoactive interfaces offering a new rout for wire-free, self-powering autonomous retinal prostheses. Photovoltaic polymers and quantum dots (QDs) were proposed as a photoelectrical retinal interface (Bareket-Keren and Hanein, 2014; Gautam et al., 2014; Antognazza et al., 2016; Maya-Vetencourt et al., 2017). QDs are s semiconducting nanometer-size crystals (typically 2–6 nm in diameter) with size-depended optical and electrical properties due to the quantum confinement effect. QDs were proposed as mediators for optical neural stimulation as a temporary electric dipole moment and localized electric fields can be generated upon optical excitation. QD films (Pappas et al., 2007; Molokanova et al., 2008) or QDs directly attached to the cell membrane via antibodies or peptides (Winter et al., 2001) were utilized for activation of neurons with light. Stimulation of RGCs with a composite QD-CNT films was also reported by Bareket-Keren and Hanein (2014).

The composite films were realized through covalent conjugation of CdSe/CdS core-shell semiconducting nanorods (NRs) to CNTs via a plasma polymerized acrylic acid layer (Bareket et al., 2017). It was reported that the NR geometry supports effective transduction of the light to charge-separation at the NR–CNT interface. The highly porous 3D CNT film enables high loading of the light-sensitive semiconducting nanocrystals. Moreover, CNTs contribute to enhancement of the electrochemical properties of the interface and its coupling to neurons due to their biomimetic structure. The electrical response of the film was typified by a voltage increase during photoexcitation followed by a slow discharge as the light was turned off (Bareket-Keren and Hanein, 2014). This kinetics was attributed to the capacitive nature of the films, charging and slowly discharging upon application and removal of the illumination stimulus. Finally, embryonic chick retinas lacking developed photoreceptors were successfully stimulated using pulsed light at a wavelength of 405 nm and ambient light intensities. The retina explants were placed on the NR–CNT composite electrodes with the RGC layer facing down (as in an epiretinal configuration). The array was used for simultaneous optical stimulation and extracellular recording. Finally, *in vitro* tests further confirmed biocompatibility and stability of the system for up to 21 days.

These results demonstrate the potential of QD based systems for autonomous photovoltaic retinal implants, and toward mimicking the activity of the damaged photoreceptors. Challenges that need to be overcome for realizing a retinal prosthesis based on such photoelectrical interfaces include the efficiency and speed of the photoelectrical response, and the potential toxicity of the photosensitive QDs (Derfus et al., 2004; Gomez et al., 2005). Previous investigations lined toxic effects such as damaged cell morphology, metabolic activity and cell viability to the presence of QDs (Lovric et al., 2005; Chan et al., 2006). These effects are considered to result from QD composition, surface coating and nanometer size (Kotov et al., 2009).

### Living electrodes

Incorporating living cells into electrode coatings was first proposed by Ochiai et al. (1980), as means to enhance power conversion of solar panels. Recently this concept is being explored toward bridging the gap at the neuron-electrode interface (Richardson-Burns et al., 2007; Green et al., 2013a; Aregueta-Robles et al., 2014). It is expected that a functional neural network coating will enhance the interaction between electrode site and the target tissue, thus minimizing the host immune response to the implant. In addition, the close proximity of neurons synapsing target tissue should reduce the amount of charge required to elicit visual percepts.

Establishing a cell laden coating requires a material that supports cell adhesion and growth during and after implantation of the bionic device. As summarized in Table 3, several perspectives have been discussed regarding the physical and biochemical considerations for new electrode coating materials to support cellular development (Abidian and Martin, 2009; Aregueta-Robles et al., 2014). In general, key design criteria for electrode coating as cellular carriers requires materials that present encapsulated cells with native tissue stiffness. *In vitro* studies have shown that neural progenitor cells have an increased proliferation rate and a decrease in apoptosis when presented with substrates with a stiffness around 1–10 kPa (Lampe et al., 2010), which is similar to *in vivo* reported tissue stiffness (Franze et al., 2013). Ideally, the cellular carrier should provide cells growth factors and degrade in a time frame consistent with cells ability to develop into a neural network. Moreover, it should allow electrode sites to passively transfer electric charge through the coating.

**Table 3:** Materials investigated as cellular carriers for living electrode coatings.

**Material**	**Polymeric scaffolds**	**Biological hydrogels**	**Synthetic hydrogels**	**Biosynthetic hydrogel hybrids**
Tailorable stiffness			PEG (Bryant and Anseth, 2002; Mahoney and Anseth, 2006; Zhou et al., 2016) PVA (Martens et al., 2007; Alves et al., 2012; Lim et al., 2015a)	PEG-based (Almany and Seliktar, 2005) PVA-based (Aregueta-Robles et al., 2015; Lim et al., 2015b)
Control on degradation rates			PEG (Wang et al., 2003; Rice and Anseth, 2004) PVA (Ossipov and Hilborn, 2006; Lim et al., 2015a) PEG-PLA (Nuttelman et al., 2002)	PEG-Fib(Almany and Seliktar, 2005) PVA-Gel (Lim et al., 2013) PVA-Gel /Ser(Lim et al., 2015b)
Support cell growth		Fibrin (Georges et al., 2006; Ahmed et al., 2008) Collagen (Ma et al., 2004; Mao and Kisaalita, 2004; Suri and Schmidt, 2010) Alginate (Novikova et al., 2006; Banerjee et al., 2009)		PEG-Fib (Almany and Seliktar, 2005) PVA-Gel (Lim et al., 2013) PVA-Gel /Ser(Lim et al., 2015a)
Support neuronal differentiation		Fibrin+NT3 (Taylor and Sakiyama-Elbert, 2006) Collagen+NT3(Houweling et al., 1998) Agarose +NGF and laminin (Yu and Bellamkonda, 2003)		PEG-CNTF (Burdick et al., 2006) PEG-PLA +NT3(Piantino et al., 2006) +BDNF(Winter et al., 2008) +NGF(Winter et al., 2007) PEVA +NGF, NT3 (Bloch et al., 2001)
Modification with anti-inflammatory agents	PLLA/PLGA/PEDOT +DEX (Abidian et al., 2006)			Alginate+PLGA+Dex (Kim and Martin, 2006)
Topographical nerve guidance	Graphene (Li et al., 2013) PLL (Daud et al., 2012)			
Allow passive charge transfer		Alginate-based (Kim et al., 2004, 2010)	PVA-based (Green et al., 2013b) PEG-based (Winter et al., 2007)	PVA-based (Goding et al., 2017a)

*αMSH, α-melanocyte stimulating hormone; NGF, nerve growth factor; BDNF, brain derived neurotrophic factor; GDNF, glial cell-derived neurotrophic factor; NT-3–neurotrophin3; DEX; Dexamethasone; PEDOT, poly(ethylene dioxythiophene); PEG, poly(ethyleneglycol); PEG-PLA, poly(ethyleneglycol)-poly(lactic acid); PEVA; Poly(ethylene-co-vinylacetate). PVA; poly(vinyl alcohol); PLLA, poly(L-lactide); PLGA, poly(lactide-co-glycolide)*.

Developing a material that meets these key criteria requires the combination of tissue engineering approaches with coating technologies. Polymeric scaffolds can readily present topographical cues for cells to grow and bridge the neural interface and have been shown to support cell growth and differentiation through modification with growth factors and anti-inflammatory agents. However, the predefined network only allows cells to grow on the backbone of the scaffold, which spatially limits neuronal ability to establish new neural connections. In addition scaffolds including conductive materials, such as graphene, result in an electrically shorted network that limits the resolution for neural stimulation. Hydrogels as tools for tissue engineering have shown to address part of the way in meeting electrode coating design criteria. Biological hydrogels such as collagen, fibrin or alginate readily support cellular interactions as they present bioactive molecules inherent to the extra cellular matrix, however mechanical properties and degradation rates cannot be tailored due to inherent variations between batches. Synthetic hydrogels fabricated with poly (vinyl alcohol) (PVA) or poly (ethyleneglycol) (PEG) have been shown as a more suitable option due to mechanical properties can be fine-tuned, while being modified with growth factors to support cellular interactions. For instance, degradable PEG-based hydrogels have shown to support neurite outgrowth through release of ciliary-neurotrophic factor (CNTF) (Burdick et al., 2006).

**Figure 4:**
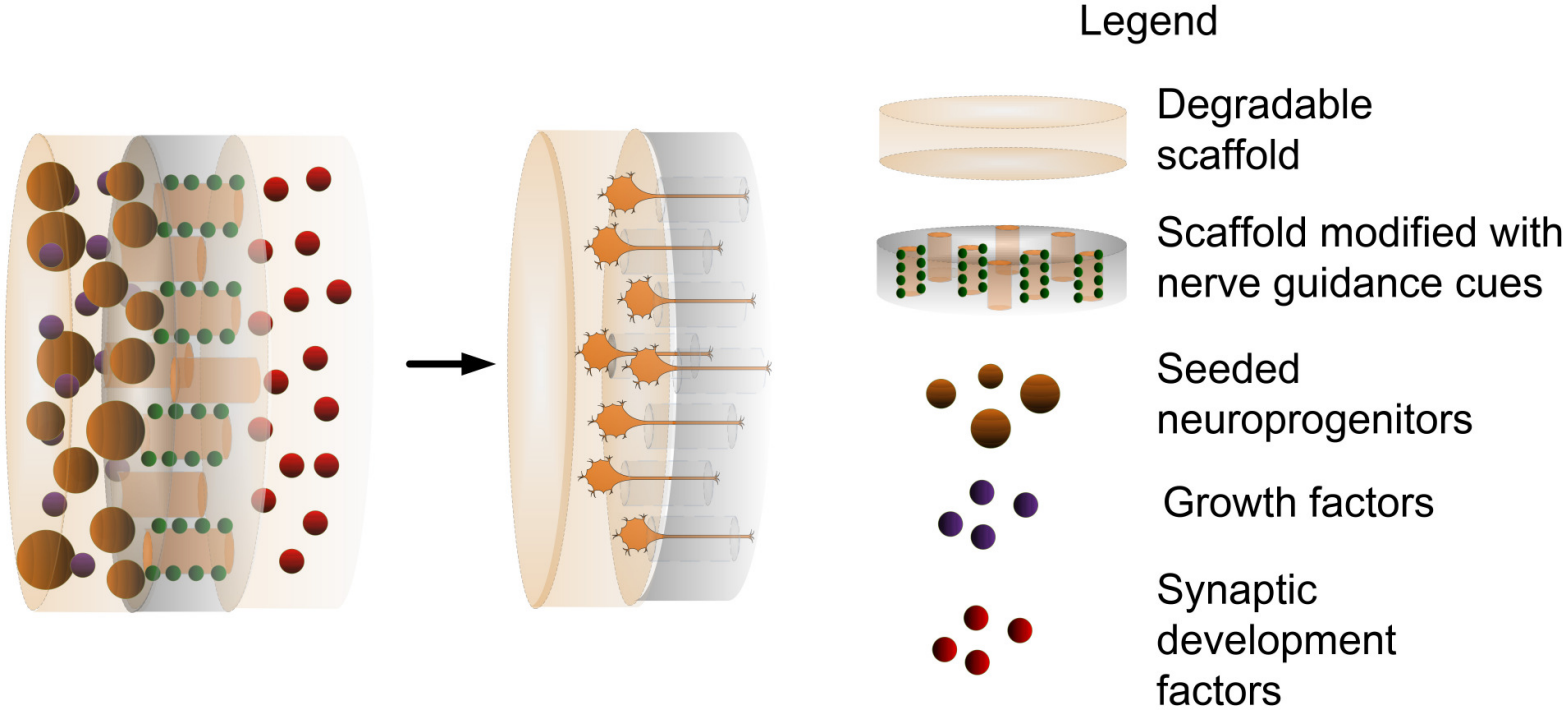
Combination of coating technologies with tissue engineering approaches as a method to bridge the neural interface.

Particularly challenging in the realm of retinal prostheses is the need for materials that allow precise control on the formation of new and stable synaptic connections. A combination of material technologies and molecules that naturally occur during nervous system development can potentially promote formation of synaptic connections between encapsulated neurons and target tissue. For instance, semaphorins (Pascual et al., 2004) are molecules that present repulsive axon guidance factors, which in developmental organisms mediate axon migration toward target tissue. Molecules such as SynCAM (Biederer et al., 2002) and Neuroligin (Scheiffele et al., 2000) are known to promote functional synapse development. Whereas, Narp (O'Brien et al., 1999) and Ephrin B1 (Dalva et al., 2000) have been identified as promoters of post-synaptic formation. Next generation retinal prostheses will include novel materials to allow a controlled spatial distribution of these molecules and promote specific connections between electrode sites and target neurons. Although it is still in early developmental stages, it is envisioned that developing such a coating could be achieved through a combination of material technologies and tissue engineering approaches as depicted in Figure 4.

### Retinal electrostimulation strategies

In physiological sight, visual stimuli are encoded by the retinal neurons in many ways. A sequence of APs is sent thought the optic nerve to different areas in the brain and midbrain to communicate the information about the visual scenes. For example, the ON and OFF pathways codify onset and offsets of light respectively. However these are not the only visual pathways from the retina; in fact, according to a recent study in mice, there are more than 32 different types of functional RGCs (Baden et al., 2016). Being able to replicate the neural code of the retina through electrical stimulation is the holy grail of bionic vision. Although, this may be an impossible goal due to the severe remodeling occurring in the retina during progression of the disease (Jones et al., 2016), research in this direction will improve restoration of visual function in those implanted with the new generations of bionic eyes. For example, Twyford et al. showed, *in vitro*, that it is possible to preferentially activate ON and OFF pathways using pulse trains at 2 kHz (Twyford et al., 2014) with their amplitude modulated. A recent modeling work by Guo et al. has also shown that by stimulating sequentially at the retina and the optic nerve it might be possible to achieve better neural encoding, as both selective activation and blockade can be exploited (Guo et al., 2017). In particular, a first stimulus was delivered in close proximity to the soma of the RGCs to activate both ON and OFF cell types. A subsequent stimulus was then delivered near the distal axon to modulate the APs previously elicited thus achieving activation of ON cells followed by activation of OFF cells. If appropriate validation of the *in silico* predictions are achieved *in vivo*, it may lead to a change in the way electrode arrays layouts are designed nowadays, as suggested by the authors.

The use of sinusoidal stimulation waveforms is becoming relevant in fields such as deep brain neurostimulation (Grossman et al., 2017). These types of waveforms are also being applied to retinal the stimulation of the retinal neurons (Twyford and Fried, 2016) to target different cells within the retinal network. In particular, Twyford et al achieved good selectivity of the RGCs at frequencies ranging 25–100 Hz (Twyford and Fried, 2016). Other researchers have used voltage-controlled Gaussian noise to analyse the filtering properties of ON and OFF RGCs (Sekhar et al., 2017). However, these studies have been performed in isolated retinae and therefore, there is still a need for validating these results *in vivo* to verify whether these responses propagate effectively to higher visual centers.

Other stimulation strategies appeared to address some of the limitations of the technology. In order to replicate the excellent results of the application of current steering techniques to cochlear neurostimulation (Firszt et al., 2007), similar approaches were adopted in the field of retinal prosthesis. Concomitant delivery of electrical stimuli using different return configurations allows for the elicitation of intermediate percepts (Dumm et al., 2014), reduction of the activation thresholds and better confinement of the electric field (Matteucci et al., 2013, 2016), and also inhibition of neural responses (Barriga-Rivera et al., 2017) among others. An increased strength of the electric field is observed when several electrodes are simultaneously activated which leads to neural inhibition. Rapid time multiplexation seems to be effective in eliciting similar cortical responses without the said inhibitory effects (Barriga-Rivera et al., 2016).

## Discussion and future lines of development

Three major implantation sites have been investigated to interface the retina and the stimulating electrode array: the epiretinal, the subretinal, and the suprachoroidal spaces (Bareket et al., 2017). Epiretinally implanted electrodes are in direct contact with RGCs thus facilitating good connection with the target cells. However, stimulation from this site is vexed by the curvature of the retina making it so that only part of the electrode array can make intimate contact with the neurons. Subretinal electrodes, implanted near the photoreceptor layer, are more prone to interact with other retinal neurons including bipolar, amacrine, and horizontal cells, thus providing poorer control over the retinal encoding. These two approaches involve higher surgical risks but better coupling with the target tissue. Nevertheless, suprachoidal electrode arrays provide a safer surgical implantation although they may already be at the limit for electrode separation. All these devices rely on metallic electrodes such as Pt, Pt alloys or gold for electrical stimulation due to their limited reactivity with tissue environment (White and Gross, 1974; Merrill et al., 2005; Polikov et al., 2005). However, the trauma from implantation along with the inherent physical and electrochemical properties of these materials induce inflammatory responses that result in electrode isolation due to fibrotic tissue encapsulation (Turner et al., 1999; Cui et al., 2003). The formation of new non-excitable tissue around the implant creates the need of increasing the charge transfer to stimulate remaining neurons. This increases the risk of damaging the tissue due to irreversible electrochemical processes. In addition, smaller and more densely packed electrode arrays are required to enhance resolution of visual perceptions (Shepherd et al., 2013). This further challenges nerve tissue stimulation due to an inherent decrease of charge transfer caused by a decrease to electrode size. Moreover, the significant mechanical mismatch between stiff materials such as Pt (~160 GPa Merker et al., 2001) and nerve tissue (<100 kPa) maintains the neural interface in a continuous inflammatory state. The latter issue further challenges retinal prosthesis as the device has to withstand ~1 × 10^5^ microsaccades per day (Yarbus, 1967) resulting in an continuous micromotion and shear stress that will persist aggravating the inflammatory response.

As described in this contribution, a way to challenge these limitations relies on coating metallic electrodes with the state-of-the-art biomaterials. While CP or CNT can help reducing the impedance of the tissue electrode interface, the body response may continue. The longevity of CPs *in vivo* remains a key challenge to be addressed. Irreversible polymer oxidation is a well-known feature of repeated cycling of electric current (Guiseppi-Elie, 2010). This leads to dopant depletion and ultimately has the effect of reducing the useful lifetime of the CP as an electrode material (Zhou et al., 2009; Balint et al., 2014). CPs may indeed have advantageous properties over metals when relatively new, but researchers working toward clinical acceptance of CPs will first have to address the important matter of longevity and sustained efficacy. Biosafety of nanomaterials is currently being investigated with numerous reports pointing to their biocompatibility. The risk of cytotoxicity was linked to properties of nanoparticles such as formulation and composition, nanometre size and surface modification potentially leading to cellular uptake and interactions with the biomolecules (Wick et al., 2007; Díaz et al., 2008; Kotov et al., 2009; Zhao et al., 2011).

The combination of these new materials with the development of new techniques that facilitate the delivery of cells within the electrodes will provide better integration between the excitable tissue and the device. While synthetic hydrogels can help to overcome some of the limitations related to the mechanical mismatch, there is still a need for connecting the stimulating elements with a source of energy and a system able to modulate the stimulating currents in order to achieve effective neural activation. In this vein, QDs and Si NWs are promising good solutions to have wire-free devices. However, as previously said, the potential cytotoxicity of some of these materials is hampering their progression to the bedside.

A relevant element in the design of retinal prosthesis is the electronic system driving the stimulating electrode array. Future retinal implants will include thousands of electrodes and these will require an electronic systems capable to activate them. In other words, each electrode needs an electronic circuit that delivers the stimulus, typically a current source. With limited space for the implant, engineers have designed systems that allow sharing a number of current sources with a subset of electrodes (Suaning et al., 2004). This is needed to minimize both space required by the electronics and the heat produced. However, visual scenes containing bright objects will require the activation of a large amount of electrodes. In this case, both spatial and time multiplexing may be required to provide a solution (Matteucci et al., 2016). This task must be performed carefully as there are neural interactions. These interactions are caused by the aforementioned crosstalk (Matteucci et al., 2016; Barriga-Rivera et al., 2017) and by facilitatory and suppressive effects produced by subsequent stimuli (Cicione et al., 2014). In addition, researchers are investigating stimulation paradigms in which the stimulus waveforms are no longer pulse-based. Instead, high-frequency pulse trains (Twyford et al., 2014) or Gaussian noise are being chosen (Sekhar et al., 2017) among others, as they are likely to produce preferential activation of different visual pathways (Twyford et al., 2014). Thus, the electronic circuitry of new generations of neurostimulators may be able to deliver arbitrary current waveforms pre-programed and adapted to the patients' needs (Samani et al., 2014). These efforts aim to make the neural messages elicited by these devices more understandable to the brain. As visual plasticity in adults is limited, pharmacological intervention may help improving the results of the rehabilitation process (Beyeler et al., 2017).

In summary, to substantially progress in the delivery of bionic vision, there is an urgent need for improving the tissue-electrode interface and the ability to selectively activate different functional types of retinal neurons. Perhaps the combination of safe implantation sites, as the case of suprachoroidal prosthesis, with the ability of growing biological wires on novel materials to offer improved interfaces with the target neurons, may provide new generations of bionic eyes able to overcome the limitations of the current technologies.

## Author contributions

All authors drafted the manuscript. AB-R, LB, and GJS revised it critically for important intellectual content.

### Conflict of interest statement

The authors declare that the research was conducted in the absence of any commercial or financial relationships that could be construed as a potential conflict of interest.
